# Immune Cells in the Tumor Microenvironment of Soft Tissue Sarcomas

**DOI:** 10.3390/cancers15245760

**Published:** 2023-12-08

**Authors:** Enar Jumaniyazova, Anastasiya Lokhonina, Dzhuliia Dzhalilova, Anna Kosyreva, Timur Fatkhudinov

**Affiliations:** 1 Research Institute of Molecular and Cellular Medicine, Peoples’ Friendship University of Russia (RUDN University), 6 Miklukho-Maklaya Street, 117198 Moscow, Russiatfat@yandex.ru (T.F.); 2 Avtsyn Research Institute of Human Morphology of Petrovsky National Research Centre of Surgery, 3 Tsyurupy Street, 117418 Moscow, Russia; 3 National Medical Research Center for Obstetrics, Gynecology and Perinatology Named after Academician V.I. Kulakov of Ministry of Healthcare of Russian Federation, 4 Oparina Street, 117997 Moscow, Russia

**Keywords:** soft tissue sarcoma, tumor microenvironment, immune cells, tumor-associated macrophages, tumor-infiltrating lymphocytes, immunotherapy, antitumor agents

## Abstract

**Simple Summary:**

Soft tissue sarcomas represent a large heterogeneous group of malignant neoplasms that are characterized by a poor disease outcome and being poorly studied. Recently, more and more studies have been directed to the role of tumor microenvironment components in establishing the aggressive potential of the tumor. Many antitumor drugs, in addition to their direct effect on tumor cells, modify the tumor microenvironment; in particular, they act on the immune component, which may be the reason for their effectiveness in soft tissue sarcoma. Immune cells themselves are now considered as promising antitumor strategies for the treatment of soft tissue sarcomas. Understanding the mechanisms of interaction between tumor cells and immune cells in different types of soft tissue sarcomas will allow us to divide patients into those for whom immunotherapy will be highly effective and those for whom this type of treatment will be ineffective.

**Abstract:**

Soft tissue sarcomas (STSs) are a rare heterogeneous group of malignant neoplasms characterized by their aggressive course and poor response to treatment. This determines the relevance of research aimed at studying the pathogenesis of STSs. By now, it is known that STSs is characterized by complex relationships between the tumor cells and immune cells of the microenvironment. Dynamic interactions between tumor cells and components of the microenvironment enhance adaptation to changing environmental conditions, which provides the high aggressive potential of STSs and resistance to antitumor therapy. Today, active research is being conducted to find effective antitumor drugs and to evaluate the possibility of using therapy with immune cells of STS. The difficulty in assessing the efficacy of new antitumor options is primarily due to the high heterogeneity of this group of malignant neoplasms. Studying the role of immune cells in the microenvironment in the progression STSs and resistance to antitumor therapies will provide the discovery of new biomarkers of the disease and the prediction of response to immunotherapy. In addition, it will help to initially divide patients into subgroups of good and poor response to immunotherapy, thus avoiding wasting precious time in selecting the appropriate antitumor agent.

## 1. Introduction

Soft tissue sarcomas (STSs) constitute a heterogeneous group of malignant neoplasms of mesenchymal or neuroectodermal origin. The masses can be found in connective tissues of the head, neck, torso (notably in the retroperitoneal space), or limbs [1]. The 2020 WHO classification recognizes over 70 histological and molecular subtypes of STS, which are clinically distinct and respond differentially to treatment [2]. The most prevalent subtypes are liposarcoma, leiomyosarcoma, pleomorphic sarcoma, and synovial sarcoma [2,3]. As these tumors are rare, their morphological verification and the choice of therapeutic tactics remain challenging. The morphological variation is complemented by diverse clinical behavior, thereby ranging from indolent progression to an aggressive, metastatic course [4,5]. Generally, the prognosis is unfavorable, with high risks of recurrence and estimated 5-year overall survival rates of 36–58% [5].

The risk factors for STSs remain understudied, although certain subtypes have been associated with environmental exposures and genetic predisposition, including neurofibromatosis and Li–Fraumeni syndrome [6]. Some STSs are age-group specific, e.g., liposarcoma in adults and rhabdomyosarcoma in children, or they typically arise in particular body regions, e.g., liposarcomas in lower limbs and synovial sarcoma, epithelioid sarcoma, and fibrosarcoma in upper limbs [7]. The rarity and morphological heterogeneity of STSs makes differential diagnosis challenging even for an expert pathologist [8]. Apart from routine histology and immunohistochemistry, the diagnostic procedure should involve molecular tests—genomic, epigenomic, and transcriptomic—to account for subtype-specific clinical patterns, which can be disease- or therapy-related [9].

STS cells continuously interact with their microenvironments [10,11,12], which contain lymphocytes and macrophages, fibroblasts, vascular beds, and the extracellular matrix. TMEs are naturally driven to suppress the antitumor immunity, as indicated by the scarcity of CD8+ T cells and the abundance of Tregs and CD206+ macrophages producing high amounts of TGFβ and IL10 [13]. Critical traits of tumor behavior, including chemoresistance and metastasis, have been related to particular properties of stromal and immune components in TMEs [14,15].

Interestingly, the STS genotype determines the composition of the TME. The totality of somatic mutations constituting the tumor mutational load correlates with the tumor’s ability to induce anticancer immunity and, moreover, its response to antitumor agents. Currently, the identification of molecular alterations that are specific to histologic subtypes of STS is a significant contribution to the definition of each individual entity [16]. Genetically, STSs include tumors characterized by specific translocations that become the driving force for tumor pathogenesis and accurate diagnostic markers of tumors with simple karyotypes; and tumors that exhibit complex genomic alterations (copy number changes, mutations in canonical drivers, and being generally characterized by deep genetics and chromosomal instability)—with complex STS karyotypes. From the immunogenomic point of view, only sarcomas bearing complex karyotypes are characterized by abundantly immune-infiltrated TMEs, i.e., they are “immune hot”, which can probably serve as a predictor of a good response to immunotherapy [16,17].

Due to the high heterogeneity of this group of malignant neoplasms and the rarity of occurrence, information on the components of TME STSs is limited. This review focuses on immune cells in TME STSs and their role in progression and resistance to antitumor therapy, as well as the potential use of immune cells for the treatment of STSs.

## 2. Tumor-Associated Macrophages in STSs

Tumor-associated macrophages (TAMs), which are an essential component of TMEs, play decisive roles in tumor-associated inflammation [18,19,20]. Under the influence of TMEs, TAMs polarize as either antioncogenic M1s or pro-oncogenic M2s [21]. M1 macrophages present antigens, produce nitric oxide and interleukins, activate T cell type I responses, inhibit cell proliferation, and promote tissue damage [22,23,24]. TAMs are implicated in angiogenesis and lymphangiogenesis, which provide the structural basis for tumor advancement and metastasis [25,26] and are considered to be putative therapeutic targets in STSs. Macrophage polarization is induced by cytokines and chemokines produced by other immune cells [27,28]. Although TMEs most definitely promote and harbor macrophage polarization [29], the exact molecular mechanisms of the interaction between STS cells and their TAMs remains obscure. Myeloid cells, which are the most prominent immune cell population within sarcomas and some epithelial tumors, differentiate into TAMs. These cells may initially have antitumor properties; still, the influence of TMEs drives them towards immunosuppressive phenotypes supporting progression of the tumor [30]. As a result, TAMs promote angiogenesis and release metabolites that feed the emerging therapy resistance mechanisms [31]. The impact can be reduced by blocking the myeloid cell recruitment to the tumor; for instance, the inhibition of the CSF1/CSF1R myeloid recruitment signature expressed by both myeloid and tumor cells was shown to effectively prevent the recruitment and survival of TAMs in carcinomas [32]. Pexidartinib, an anti-CSF1R inhibitor, has recently proved to be clinically efficacious in multiple solid tumors [33]. According to single-cell RNA sequencing data, sarcoma cells release MIF to adversely affect the activation status and protumorigenic potential of macrophages via CD74 [14]. This finding identifies the MIF/CD74 axis as principal for TME composition. Indeed, suppression of the MIF in sarcoma cells improved the secretomic profiles of tumor-infiltrating macrophages while increasing the rates of tumor antigen presentation [30]. The abundance of M2 macrophages in a sarcoma, e.g., high counts of CD163+ or CD68+ cells in nongynecological leiomyosarcomas [34], has been associated with poor prognosis. In myxoid liposarcomas, total TAM counts correlate with poor outcomes, as high levels of the TAM-produced heparin-binding EGF-like growth factor (HB-EGF) activate the EGFR-PI3K/Akt pathway in tumor cells, thereby increasing cellular plasticity and promoting chemoresistance [35]. Of note, the averaged CD163+ TAM counts correlate positively with the patient’s age and negatively with CD3+, CD4+, and CD8+ lymphocyte counts within the tumor [36]. Several studies emphasize the correlation of cellular composition of the infiltrate with the prognosis. Thus, sarcomas with high counts of CD8+ T cells in the infiltrate and which are also positive for M1 macrophages and plasma cells had better outcomes [37,38,39,40,41]. High CD20+ lymphocyte counts correlating with the rates of CD8+ infiltration [15] also appear to be prognostically favorable [13,41]. By contrast, high counts of M0 and M2 TAMs in TMEs correlate with lower CD8+ T cell counts and aggressive course [42,43].

## 3. Tumor-Infiltrating Lymphocytes in STSs

The lymphocyte infiltration rates in STSs are relatively low as compared with melanoma, renal cell carcinoma, and some other tumors [44]. The infiltrating CD3+ T cells in STSs are mostly CD8+ cytotoxic with admixtures of CD4+ T helpers and FOXP3+ Tregs. The rates and structures of lymphocyte infiltration in STSs are subtype dependent: the tumor-infiltrating lymphocyte (TIL) counts in leiomyo- and myxofibrosarcomas significantly exceed those in synovial sarcomas [44], while alveolar STSs, as well as rhabdomyosarcomas and sarcomas with generally high mutagenicity, are specifically enriched with CD3+ T cells [45,46,47]. The CD8+ cytotoxic T cell counts in the infiltrate correlate with disease severity [48]. The highest CD8+ cell counts are encountered in large masses and TMEs of metastatic tumors [49]. The constant exposure of these cells to tumor antigens and inflammatory mediators gradually exhausts their cytotoxic capacity. The exhausted effector T cells express the inhibitory receptors PD-1 and LAG3 [50]. In some non-STS tumors, TAMs are thought to inhibit the cytotoxic and chemotactic effects of CD8+ T cells [51], whereas in undifferentiated sarcomas, the CD8+ and TAM counts may correlate positively [52]. The CD4+ helper T cells exert antitumor effects by augmenting the cytotoxic T cell functionalities and clonal expansion [53,54]. Although T helper type 1 responses in STSs are considered to be weak [55], the CD4+ TIL counts may exceed those of CD8+, e.g., in differentiated or dedifferentiated retroperitoneal liposarcoma [56]; of note, CD8+ TILs in such TMEs tend to be PD-1+ exhausted.

Correlations of the infiltrating T cell counts with the prognosis in STSs are controversial. Some data suggest a beneficial influence of lymphocyte infiltration, particularly with CD4+ T cells [40,41,57,58]. The quantitative prevalence of TAMs over TILs in STSs has been associated with increased risks of metastasis [59]. Other studies, however, demonstrate a negative prognostic value of TIL counts [60] or a lack of significant association [52,61]. The inconsistency may reflect technical limitations, most notably through the use of different antibody panels with varying sensitivity thresholds [48]. In addition, the relative counts of TIL subpopulations in STSs are modulated by the treatment [62,63] and, finally, all studies enroll small cohorts due to the rarity of the tumors.

## 4. Treg Cells

The regulatory T cells (Tregs) mitigate immune responses, thereby supporting inflammatory homeostasis. Tregs produce immunosuppressive cytokines (IL-10, TGF-β), express coinhibitory molecules (CTLA-4, PD-1, PD-L1), and capture the Th1 cytokine IL-2, thereby inhibiting T cell-mediated responses and promoting immune escape of the tumor [64]. About 75% of STSs have a prominent Treg component in the infiltrate [49]. Mice with experimentally induced S180 sarcomas have increased blood and splenic counts of Tregs [65]. The scarcity of specific data on Tregs in STSs excludes rigorous projections; one study identified high counts of Treg TILs as an adverse predictor in STS [66], but another study linked the same indicator to improved progression-free survival and better response to PD-1 inhibitor pembrolizumab [67]. In a study by Smolle et al. [47], high FOXP3+ cell levels were significantly associated with improved overall survival via univariate analysis. These observations are consistent with the molecular data of Bae et al. demonstrating strong intratumoral expression of FOXP3 and other immune-associated genes in patients with relatively mild courses of STS [68]. However, Tregs were the only TIL phenotype of independent negative prognostic significance for local recurrence [47], which indicates a strong local effect of Tregs, thereby suppressing the activity of TILs and thus mitigating the antitumor immune responses. Other studies, however, revealed no significant associations of Treg counts with the prognosis in STS [40,57].

A comparative transcriptomic study on myxoid liposarcoma vs. undifferentiated pleomorphic sarcoma revealed higher expression levels of the T cell markers 4-1BB/TNFRSF9 and OX40/TNFRSF4 in the latter [69]; both 4-1BB and OX40 proteins have been characterized as Treg markers and putative targets for immunotherapy, but they are are thought to define distinct immunological profiles in STS. The OX40+ Treg cells can arrive from tertiary lymphoid structures or differentiate independently from CD4+FOXP3- T helpers inside the tumor.

## 5. B Cells

B lymphocytes have been implicated in anticancer responses [70], but their presence in STS varies; for example, in differentiated and dedifferentiated retroperitoneal liposarcomas, these cells are scarce [56]. For STSs with broad resection margins, high counts of B cells can be prognostically favorable [61]; in such cases, the cells can be nonuniformly distributed across the tumor, with entire regions being B-negative [71].

Petitprez et al. [15] analyzed in an integrative manner the influence of B cell infiltration on survival and immunotherapy response in STS, thereby confirming the positive prognostic value of this parameter, including the PD-1 inhibition sensitivity. A positive correlation between B cell marker expression and response to immunotherapy in STS was also revealed by Helmink et al. [72]. Nyström et al. [59] identified CD20+ B cells in 14% of STSs, including high-grade leiomyosarcomas, liposarcomas, and synovial sarcomas. The CD20+ infiltration rates positively correlated with survival without clear association with other prognostic factors. A recently identified subset of regulatory B cells (Bregs) was implicated in confronting inflammation and autoimmune diseases [73]. The CD1d^hi^CD5+CD19+ Bregs are thought to suppress immune responses during cancer immune surveillance by releasing anti-inflammatory mediators (IL-10) and expressing (co-) inhibitory molecules (PD-L1) [74]. Breg cells act on dendritic cells [75], macrophages [76], and Tregs [77]. Tumor-evoked CD19+CD81+CD27+pSTAT3+ regulatory B cells producing IL-10 and TGF-β suppressed T cell responses in the spleen and supported tumor growth in experimental fibrosarcoma [77].

## 6. NK Cells

The natural killer (NK) cells destroy cancer cells, and are recognized as such, and thereby participate in the antitumor immunity [78,79]. NK cells were identified in highly and moderately differentiated retroperitoneal liposarcomas using flow cytometry [56]. Some studies associate the NK TIL counts with a milder clinical course in STSs [40,80]. Compared with CD8+ cytotoxic T cells, NK cells exert a higher cytolytic activity depending on the balance of activatory and inhibitory surface molecules [81]. In GIST, NK cells express decreased levels of their specific activatory receptor NKp30/NCR3 both inside the tumor and at the periphery compared to circulating NKp30+ NK cells of healthy donors, despite the similarity of NK cell blood counts in both groups [82]. Accordingly, NK cells can be considered as an autologous tool for cytolytic immunotherapy involving specific reprogramming of their activatory receptor signatures. Thus, the rates of lymphocyte infiltration, TIL population ratios, and their roles in STS progression are tumor subtype-specific. Particular subsets of these cells can be considered as putative therapeutic targets in STSs.

## 7. Anticancer Therapies in STS: What Is in the Arsenal of Oncologists Today?

The vast number of morphologic subtypes of soft tissue sarcomas causes different response to antitumor therapies. Today, the effects that antitumor drugs have on tumor cells of STSs have been comprehensively described. However, there is very little information concerning the effects on the tumor microenvironment components, in particular on its immune component. We have described above the role of immune cells of microenvironment in carcinogenesis and progression of STSs. This section of the article is devoted to the description of the most frequently used anticancer agents for the treatment of this nosology. In Table 1, we have attempted to summarize the effects of antitumor agents on immune cells in the microenvironment. Perhaps, such a detailed approach to analyzing the role of immune cells in the microenvironment and the effects of therapeutic agents will help to explain the fact that, in some patients, the drugs give good results, while in others, there is a rapid progression.

As a rule, surgical treatment remains the standard for localized forms of STS, with adjuvant radiation therapy administered at high risks of recurrence [100,101]; the indications include tumor size > 5 cm, G2–3, and surgical margins that are positive or close (≤10 mm) [102]. Clinical decisions on adjuvant and neoadjuvant chemotherapies in STSs should be issued by multidisciplinary boards at reference medical research hospitals specializing in sarcomas. The major indications for chemotherapy in STSs include highly malignant or chemotherapy-sensitive morphological subtypes and high risks of relapse or metastasis; the decision should also account for tumor size and localization [102,103]. Pre-operative neoadjuvant chemotherapy increases the chances of R0 surgery and improves progression-free survival and quality of life after surgery.

Cytotoxic chemotherapy remains the option of choice for advanced STSs, particularly the first-line anthracycline-based schemes in locally advanced unresectable/metastatic STSs [104]. As with other modalities, the decision should account for tumor localization and morphological subtype, the patient’s age and treatment history, and metastatic status. Similarly, with localized STSs, the tactics are subject to interdisciplinary consideration at the reference medical research institutions and mandated for sarcoma management. Combined regimens of doxorubicin-ifosfamide, doxorubicin-dacarbazine or gemcitabine-docetaxel that afford better progression-free survival and higher rates of objective response compared to monotherapies are generally preferable in terms of rapid clinical benefits and local control achievement. Anthracyclines are the major class of anti-STS drugs. Cumulative anthracycline doses exceeding 550 mg/m^2^ are allowed in cases of progressive clinical and objective responses under close monitoring of the condition and observation by cardiologist. Over the last four decades, none of the other antitumor drugs outperformed anthracyclines as a first-line option in STSs [105]. In 2015, the U.S. Food and Drug Administration (FDA) approved trabectedin in unresectable/metastatic liposarcoma and leiomyosarcoma, for those previously receiving anthracyclines [106], or as first-line therapy in patients with contraindications to anthracycline treatment [106]. The approval was based on the outcomes of an ET743-SAR-3007 randomized phase III clinical trial (ClinicalTrials.gov; NCT01343277) using 1.5 mg/m^2^ trabectedin intravenously by continuous 24 h infusions for 3 weeks with 1000 mg/m^2^ dacarbazine administered intravenously once every 3 weeks [107]. A distinctive feature of trabectedin is its large number of effects on TME sarcomas, primarily its cytotoxic effect on mononuclear phagocytes. Quite unexpectedly, monocytes exposed in vitro to trabectedin were found to be severely affected and rapidly underwent apoptosis within 24–48 h. This cytotoxic effect was highly selective against monocytes and macrophages, as neutrophils or T lymphocytes were not affected. In addition, the in vitro administration of trabectedin reduced the production of CCL2, CXCL8, IL-6, VEGF, and PTX3 using primary tumor cultures and/or myxoid liposarcoma cell lines [108]. Study [109] reported that trabectedin reduced the expression of extracellular matrix-related genes produced by TAMs and fibroblasts, such as collagen type 1, fibronectin, osteopontin, and matrix metalloprotease-2 (MMP2). All these effects of trabectedin on immune cells and the extracellular matrix are of interest because they indicate that trabectedin may influence matrix remodeling. Second-line eribulin monotherapies have also been found to be specifically efficacious in leiomyo- or liposarcomas [110].

**Figure 1:**
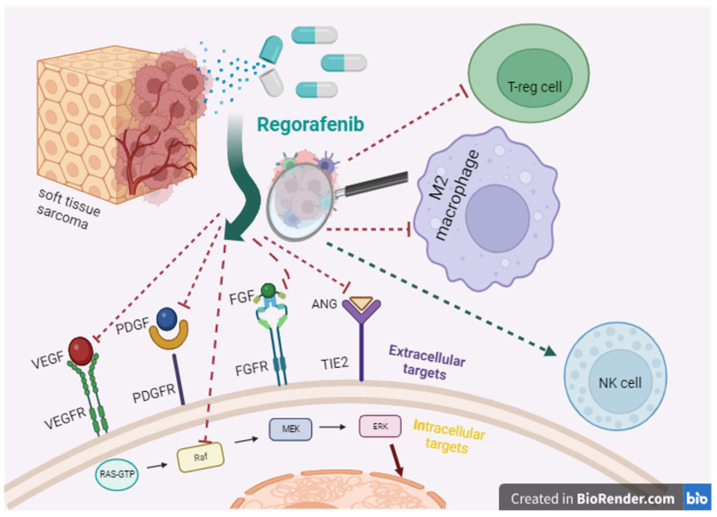
Regorafenib’s points of application.

The use of chemotherapy drugs for STSs is accompanied by high toxicity, and the response is temporary. Much-anticipated targeted options emerged as a result of extensive research on the regulatory pathways involved in sarcomagenesis. Imatinib mesylate, a tyrosine kinase inhibitor, is effective against GIST. About 75% of these tumors harbor activating mutations in Kit [111]. Imatinib mesylate inhibits both Kit and PDGFRα, thus affording partial response or stabilization in >80% of recipients with advanced GIST; the targeted strategy increased median survival from 1 to 5 years since commencement [112]. The effect of imatinib on STSs TMEs is associated with an increase in the ratio of effector T cells to Tregs, which activates the antitumor immune response [113]. Pazopanib is an orally delivered small molecule inhibitor of multiple receptor tyrosine kinases, including VEGFR-1/2/3, PDGFR-α/β, c-Kit, FGFR-1/3, and c-Fms [114]. Pazopanib is efficacious in all nonadipose-derived STSs [115]. The drug is used as a >2nd-line option in patients with disseminated STS previously receiving anthracyclines (except liposarcoma cases). In certain rare STS subtypes, pazopanib can be efficacious as a first-line treatment. Regorafenib is another kinase inhibitor that blocks the activity of several receptors; the biological effects include angiogenesis inhibition and TME remodeling [116]. Regorafenib has shown efficacy in metastatic STSs, including angiosarcoma, synovial sarcoma, leiomyosarcoma, and other histological subtypes, except liposarcoma [117]. Regorafenib is interesting because in addition to acting on a large number of targets on cancer cells, it also has an effect on immune TME cells [94,95,96], (Figure 1).

There is a relationship between angiogenesis in tumor and adaptive immunity. Thus, the suppression of tumor neoangiogenesis using antiangiogenic drugs is accompanied by the migration of T cells into the tumor and the limitation of immunosuppression mechanisms. Antiangiogenic drugs, by affecting the TMEs of STSs, transform the TMEs from “cold” to “hot” [16]. Taking into account this property of antiangiogenic agents, the combination of antiangiogenic drugs with ICIs for the treatment of several types of STSs is being actively studied [118,119].

Gene fusions involving the NTRK family result in the constitutive activity of TRK receptor kinases. The fusion proteins facilitate oncogenesis through PI3K/AKT, RAS/MAPK/ERK, and PLCγ pathway activation [120]. A TRK inhibitor larotrectinib is efficacious in NTRK-rearranged STSs, with an overall response frequency of 75% [121]. Crizotinib therapy is indicated in ALK-rearranged myofibroblastic sarcomas [122]. The use of palbociclib, a CDK-4/6 inhibitor, can be considered in patients with high- or low-differentiated liposarcoma [123].

**Figure 2:**
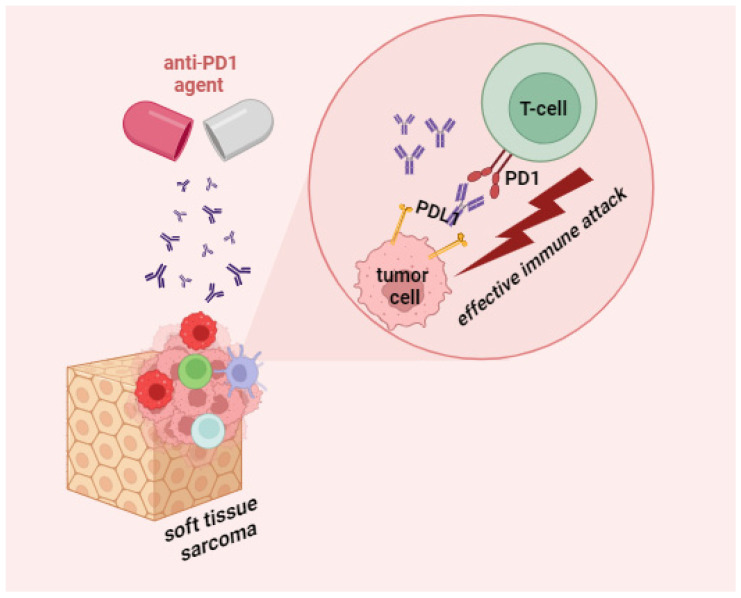
Mechanism of action of anti-PD1 agents in STS.

Immunotherapy for STS is a complicated issue, the history of which can be traced to the cornerstone experiments by William Coley, who treated limb sarcoma with intratumor injections of modified bacteria; the resulting inflammatory reaction to the pathogen promoted the regression of the tumor [124]. Despite the long history, current prospects of immunotherapy for STS leave much to be desired because of the high subtype variation and chaotic antigenic landscapes. For liposarcomas and undifferentiated pleomorphic sarcomas, the best studied STS subtypes, the response patterns are relatively promising. PD1/PDL1 inhibiters unblock the immune suppression of antitumor T cells, which results in T cell multiplication and permeation into the TME and inducing an antitumor response (Figure 2).

For liposarcomas and undifferentiated pleomorphic sarcomas, the best studied STS subtypes, the response patterns are relatively promising. Leiomyo- and synovial sarcomas are invariably resistant to immune checkpoint inhibitors, whereas alveolar STSs are particularly sensitive [105]. A survey of nine studies on anti-PD1/anti-PDL1 options in STSs revealed an overall response frequency of 15.1%: 18.7% as a monotherapy and 13.4% in combination with other immunotherapy drugs or antiangiogenic agents [125]. Meta-analyses [126,127] revealed a negative association between PD-L1 overexpression and survival in STSs, which sparked the interest in corresponding immunotherapies. Pembrolizumab was clinically efficacious in an SARC028 trial (2017) enrolling patients with undifferentiated pleomorphic sarcoma and dedifferentiated liposarcoma [128]. Sarcoma immune class (SIC) classifications of patients confirmed that tumors in the immune high class (SIC E) showed the most benefit in response to pembrolizumab [15]. In the search for an effective antitumor drug combination, several studies have attempted to combine pemprolizumab with doxorubicin (NCT02888665) [129] and axitinib (VEGFR-inhibitor targeting drug) (NCT02636725) [118]. In these studies, longer progression-free survival was achieved in patients with high TIL density in the tumor and high PD-L1 expression; thus, a better response was achieved in sarcoma patients with a “hot” immune phenotype. Nivolumab (a PD 1/PD L binding inhibitor) and ipilimumab (a T cell CTLA 4 inhibitor) were preliminarily evaluated as efficacious in undifferentiated pleomorphic sarcoma, myxofibrosarcoma, leiomyosarcoma, and angiosarcoma through the results of an Alliance A091401 trial (2018) [130]. However, the response frequency for immunotherapy in the STSs never exceeded 30%. The PD-L1 expression is considered to be prognostically favorable in many cancers, but for STSs, its value is nonuniform [131], and in most STS subtypes, the PD-L1 index is low (10–22%) [45,132].

Given the high mutation burden and immune cell infiltration in STSs, the quest for agonists and markers of immune response to these cancers remains a challenge.

## 8. Opening New Horizons or Promising Immunotherapeutic Options of STS

### 8.1. The Therapy Targeting TAMs (Tumor Associated Macrophages)

As it was mentioned earlier, TME STS is characterized by the predominance of M2-type macrophages, which secrete a large amount of protumor cytokines, thereby contributing to angiogenesis and the progression of STS. Taking into account the pro-oncogenic potential of M2 macrophages, the search for options aimed at blocking the polarization of macrophages in this direction or their conversion to the M1 state has been actively conducted. In a preclinical study, mice with sarcomas were injected with calcium zoledronate nanoparticles (CaZol@pMNP), which led to a significant reductions in tumor growth [133]. Zoledronate (nitrogen-containing bisphosphonate), in turn, has cytotoxicity towards TAMs [134], but its short half-life is an obstacle to its use in this direction. Nanoparticles were used to prolong the circulation of zoledronate and to achieve a suppressive effect on TAMs. In a mouse model with S180 tumors, CaZol@pMNP effectively depleted TAMs [133]. In another study [135], Ang-2/VEGF-neutralizing peptide antibody AS16-Fc was shown to reduce the polarization of M2 macrophages, in addition to its direct inhibitory effect on angiogenesis in an S180 mouse sarcoma cell line. In the course of testing TLR2 agonist, the researchers managed to obtain macrophages with a pronounced antitumor potential and achieved a significant increase in the M1/M2 ratio in sarcoma mice in sarcoma mice [136]. Another important signaling pathway is CD47-SIRPα; it allows tumor cells to escape immune killing by suppressing the phagocytic ability of macrophages. The study (NCT02890368) testing TTI-621 was shown to have an affinity for SIRP1α and demonstrated the inhibition of the binding of CD47 to SIRP1α, which activates macrophage phagocytosis [137]. TTI-621 exhibits antitumor activity in mice with STS. A synthetic agonist of Toll-like receptor 4 (TLR4), an activating receptor expressed by macrophages and other innate immune cells, is in phase I development for the treatment of metastatic sarcoma. The study is investigating this agent as an additive component that enhances tumor antigen release to radiation therapy (NCT02180698) [138]. One of the promising strategies to alter TAM polarization is the use of N-methyl-D-aspartate ion channel receptor (NMDAR) antagonists. NMDAR activation triggers calcium influx and reactive oxygen species production, which stimulates the immunosuppressive activity of TAMs. The NMDAR antagonists MK-801, memantine, and magnesium effectively inhibit these processes in TAMs. Single-cell RNA sequencing analysis showed that blocking the NMDAR promotes the change of the TAM phenotype into an antitumor phenotype [139]. Studies aimed at controlling TAM polarization are ongoing. Given the important role of M2-type macrophages in creating an immunosuppressive environment in TME STSs, switching macrophages from a protumor type to an antitumor state remains a promising strategy for cancer immunotherapy.

### 8.2. Helping “Their Own” or Tumor-Infiltrating Lymphocyte Therapy

Based on the fact that the high infiltration of TILs is associated with favorable outcomes in STS, their use as antitumor agents is being actively investigated. For such therapy, as a rule, TILs are extracted from the tumor and, after ex vivo cell multiplication, are injected back to the patient [140]. When tumor-specific TILs are cultured ex vivo, away from the suppressive TME, the balance shifts in favor of tumor-reactive lymphocytes. In 2021, a protocol to obtain TIL from resected STS cells was first presented by Mullinax et al. [141], which initiated a study (NCT04052334). A significant deterrent to the introduction of TIL into clinical practice is the high cost of such therapy due to its personalized nature.

### 8.3. Using T Cells for Treatment of STS

One of the causes of suppressed antitumor immunity in STSs is the impaired recognition of cancer antigens by T cells. Therefore, the main point of T cell therapy is to enhance antitumor immunity by delivering modified T cells. Autologous T cells are obtained from the patient’s peripheral blood or from the primary tumor and then amplified. Under ex vivo conditions, T cells are “tuned” against an antigen that is expressed by tumor cells so that the T cells acquire the ability to “recognize the enemy”. The main approaches are T cell therapy with engineered T cell receptors (TCRs) and chimeric antigen receptors (CARs). CARs are chimeric antigenic receptors that are artificially created to recognize naturally occurring tumor surface antigens and activate T cells in an MHC-independent manner [142]. A study [143] tested the efficacy of HER2-CAR T cell therapy for patients with refractory metastatic sarcoma. Overall, survival after 1 year was 60% for patients treated with HER2-CAR T cell therapy and lymphodepletion with fludarabine or cyclophosphamide [143]. Despite this good result, further studies on the efficacy of this therapy for patients with STSs are needed, as the patient sample in the above-mentioned study was small and heterogeneous.

STS, as well as many other solid tumors, is characterized by the expression of the testicular cancer antigens (CTA)-MAGE and NY-ESO-1 (tumor-associated antigens). Since CTA epitopes are recognized by autologous T lymphocytes that target cancer cells, they are considered as therapeutic targets in a number of malignancies [144]. An increased degree of CTA expression has been observed in different subtypes of STS, such as synovial sarcomas and myxoid/round cell liposarcomas, angiosarcoma, malignant fibrous histiosarcoma, and chondrosarcoma [145]. From a prognostic point of view, it has been shown that the increased expression of NY-ESO-1 in STS acts as an unfavorable prognostic factor. Increased immunohistochemical expression of NY-ESO-1 was significantly correlated with tumor size, advanced stage, and poor prognosis of the disease [146]. The expression of these antigens was found in more than half of primary synovial sarcoma samples [147]. In a pilot study using T cells engineered to express a recombinant TCR that specifically targets NY-ESO-1, combined with IL-2 infusion, this resulted in objective clinical responses in more than half of patients with refractory and metastatic synovial sarcoma [148]. The authors state that circulating CD8+ T cells against NY-ESO-1 retain their functional activity for several months after infusion to the patient. A detailed description of NY-ESO-1 as a diagnostic marker and a target for additive therapy of STS is presented in the manuscript by Smith and Iwenofu [149]. Studies evaluating the efficacy of TCRs targeting MAGE for the treatment of STS (NCT04044768) [150], (NCT03132922) [151] are actively conducted.

### 8.4. Using NK Cells for Treatment of STS

The proper functioning of NK cells plays a key role in cancer immune surveillance, thereby prompting researchers to utilize NK cells for the treatment of STS. The effects of administering NK cells to patients with STSs to enhance the antitumor immune response are being investigated. The study [152] (NCT02890758) is evaluating the use of universal donor NK cells for patients with a variety of cancer indications, including STS. Typically, HLA-matched donor NK cells are used for NK cell therapy. This study utilizes healthy donor NK cells in combination with ALT803, a protein that keeps NK cells growing and viable for a long time. A combination of expanded NK cells and radiation therapy has proven to be effective in a first-in-dog clinical trial, thus showing synergy between radiation therapy and expanded NK cells. RT increased sarcomas’ susceptibility to NK cell cytotoxicity and improved the tumor homing of adoptively transferred cells, thereby providing a rationale for testing such combinations in clinical settings [153].

### 8.5. Dendritic Cells for the Treatment of STS

The direct delivery of antigen-presenting cells as part of a cell-based vaccine for the treatment of STSs is also being explored. Autologous dendritic cells exposed to tumor-associated antigens or whole tumor cell lysates is a strategy that has also been tested in STSs [154,155]. An ongoing phase I study is investigating a monocyte dendritic cells vaccine in pediatric patients with advanced sarcoma, including (EudraCT 2014-003388-39). After such treatment, an enhanced antitumor response of T cells ex vivo to this kind of antigen-presenting cell therapy was revealed [156].

## 9. Conclusions

STSs constitute one of the least studied groups of malignant neoplasms. Advances in their diagnosis and therapy are dependent on basic research investigating the role of the tumor microenvironment. Further understanding of the interaction between tumor cells and immune cells in the TME will allow for the creation of new prognostic scales of responses to treatment and the introduction of new effective drug combinations into the arsenal of oncologists.

## Figures and Tables

**Table 1:** Effects of antitumor drugs on immune cells in the STS TME.

Name of the Drug	Action on the Immune Component of the STS TME
Doxorubicin	✓ Causes an antitumor response by inducing a CD8+ T-cell immune response [83]. ✓ Induces the up-regulation of immune-related genes of the PD-1/PD-L1 [84]. ✓ Promotes the recruitment of CCR2+ monocytic cells [85].
Trabectedin	✓ Suppresses the production of cytokines and chemokines secreted by monocytes/macrophages [86]. ✓ Can inhibit the differentiation of monocytes into macrophages. ✓ Reduces the production of two proinflammatory mediators, CCL2 (responsible for recruiting monocytes to tumor sites) and IL-6 (tumor growth factor), by monocytes and TAMs [87].
Ifosfamide	✓ Dose-dependent reduction of T cells [88].
Gemcitabine	✓ Induces a decrease in the infiltration of CD8+ and CD4+ T cells [89]. ✓ Leads to an increase in the number of immunosuppressive MDSCs. ✓ Increases the number of M2-polarized macrophages. ✓ Reduces the concentration of regulatory T cells [90].
Docetaxel	✓ Promotes intratumor infiltration of T cells and increases PD1 and PD-L1 levels [91].
Imatinib mesylate	✓ Inhibits dendritic cell formation, thereby resulting in less efficient priming of cytotoxic cells. ✓ Promotes acquisition of M2 phenotype by macrophages [92]. ✓ Increases the number of effector T cells.
Pazopanib	✓ Enhances antitumor activity of T lymphocytes. ✓ Induces antitumor activity of NK cells. ✓ Reduces the number of Treg cells. ✓ Reduces the number of CD14+ monocytes [93].
Regorafenib	✓ Improves NK cell function. ✓ Inhibits TAM infiltration by inhibiting TIE2 pathway [94]. ✓ Causes sustained M1 polarization and prevents M2 polarization of macrophages [95]. ✓ Reduces the number of Treg cells [96].
Crizotinib	✓ Induces infiltration by CD8+ cytotoxic T cells. ✓ Increases the number of dendritic cells. ✓ Reduces the number of Treg cells [97].
Palbociclib	✓ Disrupts proliferation of activated CD3+ T cells. ✓ Reduces the number of Treg cells [98].
Pembrolizumab	✓ Increases tumor infiltration by T cells. ✓ Restores effective T cell responses against tumor cells. ✓ Inhibits negative regulation of T cell responses [99].

## Data Availability

The data can be shared up on request.
